# FAM83 proteins: Fostering new interactions to drive oncogenic signaling and therapeutic resistance

**DOI:** 10.18632/oncotarget.9544

**Published:** 2016-05-21

**Authors:** Courtney A. Bartel, Neetha Parameswaran, Rocky Cipriano, Mark W. Jackson

**Affiliations:** ^1^ Department of Pathology, Case Western Reserve University, Cleveland, Ohio, United States of America; ^2^ Case Comprehensive Cancer Center, Case Western Reserve University, Cleveland, Ohio, United States of America

**Keywords:** FAM83, EGFR, Oncogene, resistance

## Abstract

The FAM83 proteins were recently identified as novel transforming oncogenes that function as intermediaries in EGFR/RAS signaling. Using two distinct forward genetics screens, the Bissell and Jackson laboratories uncovered the importance of the FAM83 proteins in promoting resistance to EGFR tyrosine kinase inhibitors and therapies targeting downstream EGFR signaling effectors. The discovery of this novel oncogene family using distinct genetic screens provides compelling evidence that the FAM83 proteins are key oncogenic players in cancer-associated signaling when they are overexpressed or dysregulated. Consistent with a role in oncogenic transformation, the FAM83 genes are frequently overexpressed in diverse human cancer specimens. Importantly, ablation of numerous FAM83 members results in a marked suppression of cancer-associated signaling and loss of tumorigenic potential. Here, we review the current knowledge of the FAM83 proteins’ involvement in cancer signaling and discuss the potential mechanisms by which they contribute to tumorigenesis. Both redundant activities shared by all 8 FAM83 members and non-redundant activities unique to each member are highlighted. We discuss the promise and challenges of the FAM83 proteins as novel points of attack for future cancer therapies.

## INTRODUCTION

Cell signaling networks are complex, interconnected pathways that work in unison to control cellular function. When these tightly controlled pathways are disrupted, however, cells may become cancerous [1–3]. Identifying key pro-malignant signaling pathways is crucial to finding new therapeutic targets and improving survival. As major pro-malignant signaling pathways are discovered, efforts can be made to therapeutically target them, with hopes of benefiting cancer patients. Inhibitors to important signaling proteins such as receptor tyrosine kinases (RTKs; EGFR, HER2, IGFR; c-Kit, MET, FGFR), and their downstream effectors (RAS, RAF, PI3K, AKT, and mTOR) have been identified, with additional searches underway [4–16]. While these targeted, precision medicines have had some success, they are not the silver bullets they were once anticipated to be. In part, this is due to potent compensatory signaling mechanisms engaged by cancer cells to circumvent the loss of key survival signals [17–21]. While the use of current precision medicines is continually being refined by the identification of predictive biomarkers, the use of synergistic drug combinations, and the optimization of drug administration timing, it is clear that efforts towards identifying novel potential therapeutic targets should continue.

**Figure 1:**
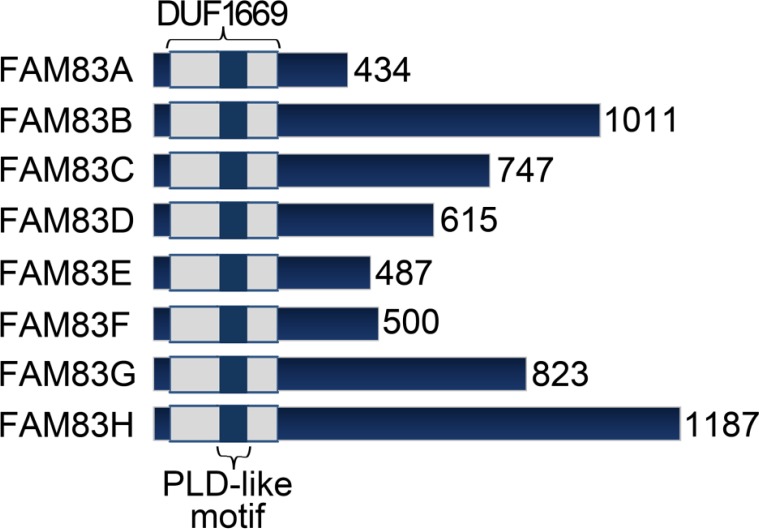
FAM83 proteins FAM83 members are characterized by a common N-terminal Domain of Unknown Function 1669 (DUF1669), but have vastly different C-terminal regions.

**Figure 2:**
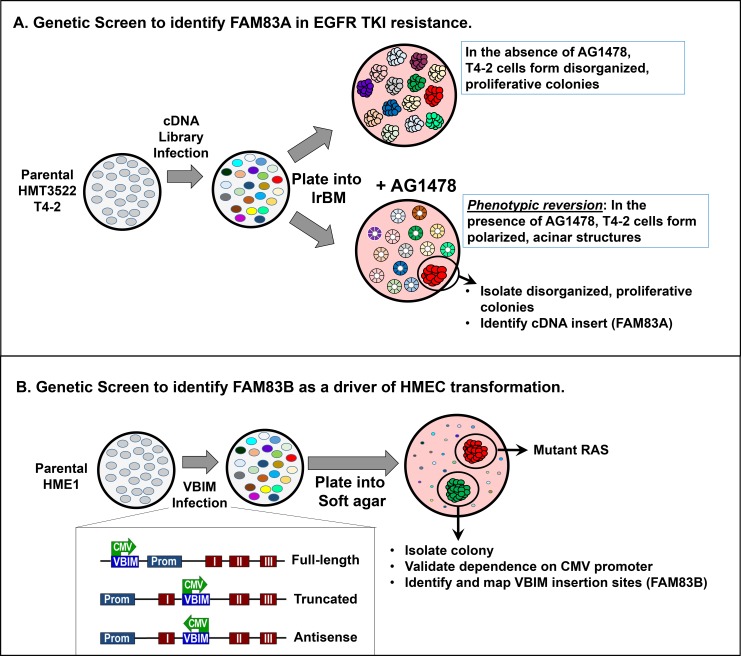
Distinct genetic screens identifying FAM83A and FAM83B **A.** The Bissell Laboratory utilized a phenotypic reversion screen to identify for genes that confer resistance the EGFR tyrosine kinase inhibitor AG1478 in breast cancer. HMT3522 T4-2 cells were infected with a retroviral cDNA library. Infected cells were grown in 3D laminin-rich basement membrane. In the absence of AG1478, all T4-2 cells form disorganized, proliferative colonies. In the presence of AG1478, the cells will “revert” to form polarized, acinar structures that resemble non-transformed cells. AG1478-resistant cells that continue to form disorganized, proliferative colonies are isolated, and the cDNA conferring AG1478 resistance was identified as FAM83A. **B.** The Jackson Laboratory utilized an insertional mutagenesis screen to identify genetic changes that promote the transformation of HMEC. Non-transformed HME1 cells (which can be transformed by a single genetic event, such as mutant RAS) were infected with Validation-Based Insertional Mutagenesis lentiviruses. Following VBIM infection and integration, a CMV promoter is randomly inserted throughout the genome, modifying transcription of nearby genes (most commonly by causing high-level expression full-length, truncated, or anti-sense mRNAs). VBIM-infected cells were plated into soft agar to identify mutants capable of anchorage-independent growth, a hallmark of transformed cells. Identified mutants were validated by adding Cre, which removes the VBIM insertion *via* loxP sites in the construct, and the VBIM insertion site was mapped to the FAM83B gene.

Here, we discuss a novel family of signaling proteins, named FAM83 (FAMily with sequence similarity 83), that function as key intermediates in oncogenic EGFR, MAPK and PI3K/AKT signaling [22–25]. Due to their involvement in a variety of important cancer cell signaling functions and overexpression in cancer, the FAM83 proteins are emerging as intriguing oncogenes worthy of additional study. There are 8 FAM83 genes, named FAM83A-H, with each located at a distinct genomic site (Figure 1 and Table 1). Each FAM83 gene encodes a protein classified solely on the presence of a highly conserved domain of unknown function (DUF1669) located in the N-terminus. Evolutionarily, there are no homologues or orthologues of FAM83 genes in primitive organisms such as Drosophila melanogaster, Saccharomyces cerevisiae, and Caenorhabditis elegans, but all jawed vertebrates appear to encode the FAM83 genes [26]. Interestingly, ancient vertebrates such as lampreys express FAM83B and FAM83E-H (but not FAM83 A, C and D), suggesting that these may have been the earliest FAM83 genes to appear. Until 2012, there were no reports about the transformative functions of the FAM83 proteins, but since, multiple studies have identified overexpressed or dysregulated FAM83 members as key regulators of transformation (cell growth, proliferation, and metastasis) and resistance to precision therapies [22, 25, 27]. The first detailed analysis of the FAM83 proteins in transformation resulted from the separate identification of FAM83A and FAM83B in distinct genetic screens performed by the Bissell and Jackson Laboratories, respectively. As detailed below, the Bissell laboratory identified FAM83A using a retroviral cDNA library screen for genes that confer resistance to an EGFR tyrosine kinase inhibitor (TKI) in tumorigenic mammary epithelial cells [25]. Simultaneously, the Jackson lab identified FAM83B using an insertional mutagenesis screen for genes that drive human mammary epithelial cell transformation, similarly to active RAS [22] (Figure 2). Taken together, these studies provide the first evidence that a novel family of uncharacterized proteins plays an important role in the EGFR and RAS signaling pathways and, when dysregulated, can promote malignant transformation. The separate identification of two FAM83 members in distinct genetic screens and their elevated expression in a diverse set of cancer types highlights their importance and provides a vital new avenue for potential therapeutic intervention.

**Figure 3:**
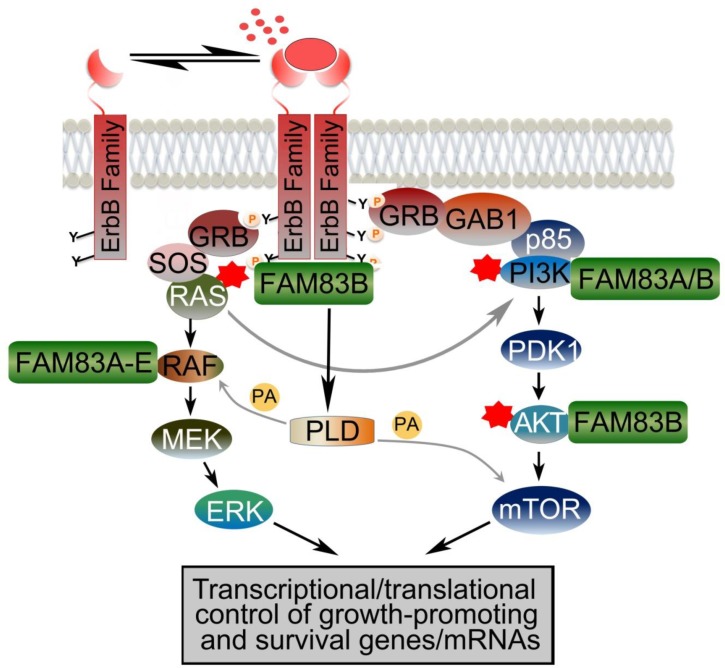
FAM83 family of proteins promotes ErbB receptor signaling The ErbB signaling network controls normal cell growth, survival and proliferation. Ligand-mediated activation of the ErbB family of receptor tyrosine kinases results in receptor dimerization, autophosphorylation of the receptors and activation of downstream signaling effectors. ErbB receptors activate RAS/MAPK and PI3K/AKT/mTOR pathways and Phospholipase D (PLD), among others. PLD generates Phosphatidic acid (PA) that enhances RAF recruitment to the membrane and also activates mTOR signaling. In many cancers, activating mutations in RAS, PI3K and AKT drive transformation by inappropriately activating the MAPK and PI3K/AKT pathways. The novel FAM83 (FAMily with sequence similarity 83) family of signaling proteins have emerged as important therapeutic targets as they are overexpressed in many cancers and they function as key intermediates in EGFR, MAPK and PI3K/AKT signaling.

One of the most promising potential roles for the FAM83 proteins is within the ErbB signaling network, both at the receptor level and the downstream MAPK and PI3K/AKT pathways. The ErbB signaling network tightly controls normal cell growth and proliferation [28]. Often, the aberrant activation of RTKs, such as the ErbB receptors, drives transformation by inappropriately activating the MAPK and PI3K/AKT pathways [29]. The ErbB family consists of four homologous RTKs: ErbB1/EGFR, ErbB2/HER2/Neu, ErbB3/HER3, and ErbB4/HER4. Ligand-mediated stimulation of the ErbB proteins results in homodimerization or heterodimerization, autophosphorylation of the receptors, and activation of downstream signaling effectors (Figure 3). ErbB receptors can lead to the activation of the RAS/MAPK and PI3K/AKT/mTOR pathways, Signal Transducer and Activator of Transcription 3 (STAT3), and Phospholipase D (PLD), among others [28, 30]. The MAPK and PI3K/AKT pathways are activated by the recruitment of adaptor proteins, such as Grb2 or Shc, which in turn recruit son of sevenless (SOS) and RAS to the receptor, resulting in RAS activation [31]. Following its activation, RAS recruits RAF to the membrane where it is activated and, subsequently, RAF phosphorylates MEK1 and MEK2, which activate ERK1 and ERK2 [32]. The ERK proteins then activate transcription factors responsible for regulating growth and proliferation. RAS activation also activates PI3-Kinase, by binding to p110 catalytic subunit, which phosphorylates phosphatidylinositol (4,5) bisphosphate (PIP2) PIP3 [33]. The formation of increased PIP3 activates Phosphoinositide-dependent protein kinase (PDK-1), and recruits AKT to the membrane for activation, which then phosphorylates and activates mammalian target of rapamycin(mTOR) [34]. Collectively, these processes regulate normal cellular function by promoting cell growth and survival in response to MAPK and PI3K/AKT pathway activation. A complete understanding of the protein complexes that regulate RTK and downstream effector activation is critical to identifying new ways to suppress their inappropriate activation in cancer.

The ErbB receptors and MAPK and PI3K/AKT signaling cascades are the subject of intense research aimed at identifying pharmacological inhibitors that will suppress growth signaling and prevent cancer cell proliferation. Precision therapies aimed at disrupting ErbB RTKs (erlotinib, gefitinib, cetuximab, lapatinib, trastuzumab, pertuzumab) [35], RAS (Farnesyltransferase inhibitors and select isoform inhibitors) [36], RAF (Vemurafenib, Dabrafenib, Trametinib, RAF265, CCT196969, CCT241161) [37, 38], MEK (AZD8330, Selumetinib, MEK162, PD0325901, Refametinib, Cobimetinib) [39], or the PI3K/AKT/mTOR pathway (Everolimus, Temsirolimus, BEZ235, GDC-0980, PF-05212384, BAY80-6946, Buparlisib, GDC-0032, Perifosine, MK2206, AZD2014, MLN0128) [40] have been developed and are currently approved for patient use or are being evaluated in a number of clinical trials [41, 42]. However, the complexity of signaling interactions limit the effectiveness of these therapies, and resistance develops easily due to multiple regulatory feedback loops that are engaged by cancer cells to circumvent the therapy-induced death or proliferative arrest [17]. For example, nearly all patients treated with the EGFR inhibitor erlotinib, including those that have a robust, immediate response, will develop resistance [43]. In fact, resistance has been observed for nearly all precision medicines when used as single agents [44]. Furthermore, combining precision therapies in an attempt to suppress the feedback mechanisms that are responsible for resistance has also proven difficult, given the toxicity associated with suppressing these key growth-regulating pathways in normal, non-cancerous cells [45]. We propose that the FAM83 proteins provide new opportunities for drug development to overcome resistance to current therapies. All FAM83 members have significantly increased expression in cancer, providing unique potential targets to specifically kill malignant cells (Table 1). In this review, we will discuss data implicating FAM83 members in the aberrant activation of EGFR, MAPK, PI3K/AKT, and other cancer-associated signaling pathways and discuss their potential to serve as novel therapeutic targets.

**Table 1:** FAM83 proteins are overexpressed in cancer

FAM83 proteins	Chromosomal location	Cancer tissue overexpression	Cancer related functions	Refs.
FAM83A	8q24.13	Breast, Ovaary, Pancreas, Bladder, Colon, Lung, Testis	Participates in EGFR and Ras signaling;promotes growth, invasiveness and transformation; TKI resistance	[24,25]
FAM83B	6p12.1	Breast, Ovary, Lung	Participates in EGFR and Ras signaling;promotes growth, invasiveness and transformation; TKI resistance	[22–24,28]
FAM83C	20q11.22	Bladder	Binds CRAF; epithelial cell transformation	[24]
FAM83D	20q11.23	Breast, Liver, Lung, Bladder, Ovary, Testis, Adrenal, Thyroid, Lymphoid	Binds CRAF; Promotes mTOR activation; Rapamycin sensitivity	[24, 79]
FAM83E	19q13.13	Ovary	Binds CRAF; epithelial cell transformation	[24]
FAM83F	22q13.1	Esophageal Squamous cell Carcinoma	Invasion, metastasis	[82]
FAM83G	17p11.2	Breast	Participates in EGF-driven Src/Akt signaling	[72, 74–75]
FAM83H	8q24.3	Colon	Supports invasion and metatasis by promoting keratin	[89]

The table highlights the distinct chromosomal locations of FAM83A-H members and summarizes their overexpression and oncogenic functions in a multitude of human cancers.

## FOCUS ON FAM83A

The smallest FAM83 protein, FAM83A (originally named BJ-TSA-9), was first implicated as a potential cancer biomarker in 2008. Liu *et al.* used nested PCR to show that FAM83A mRNA is highly expressed in the circulating tumor cells (CTCs) of lung adenocarcinoma patients [46]. When used in combination with two other known lung cancer biomarker genes, including squamous cell carcinoma antigen (SCCA) and lung-specific X protein (LUNX), the rate of circulating tumor cell detection was as high as 81.4%. Moreover, patients who had persistent expression of FAM83A, SCCA, and LUNX in CTCs were more likely to have more advanced disease and a greater risk of recurrence. In addition to identifying FAM83A as a potential lung cancer biomarker, Liu *et al.* also identified FAM83A as a potential biomarker in breast cancer [47]. Using a panel of three genes, FAM83A, NPY1R, and KRT19, the authors again used a rapid nested PCR assay to detect breast cancer cells in peripheral blood samples. When all three gene markers were combined, cancer cells were detected in the peripheral blood of 79.6% of breast cancer patients. The detection of FAM83A also correlated with the presence of distant metastases. Importantly, FAM83A was not identified in peripheral blood samples of normal, non-cancerous patient donors, implicating FAM83A as a diagnostic and prognostic cancer biomarker.

In addition to the potential use of FAM83A as a biomarker, a forward genetic screen identified FAM83A as a driver of EGFR tyrosine kinase inhibitor (TKI) resistance, suggesting FAM83A plays an important role in cancer cell biology [25]. EGFR is frequently overexpressed, mutated, or hyperactivated by excess ligand in a wide variety of cancers, including lung, brain, head and neck, and breast, among others [29, 48]. TKIs, including erlotinib and gefitinib, prevent activation of EGFR by blocking the ATP-binding pocket. When activating mutations or deletions are responsible for EGFR hyperactivation, as is often is observed in lung cancer, TKIs are often initially effective treatment options [49, 50]. In cancers where EGFR is rarely mutated, high EGFR activity can still be evident, resulting in hyperactive downstream MAPK and PI3K/AKT/mTOR signaling [51]. One example of high-level EGFR activity in the absence of mutation is the highly aggressive basal-like subtype of breast cancer (BLBC). Genomic analysis has identified EGFR-driven expression signatures in ~90% of the BLBC [52]. Importantly, EGFR expression is higher in patients harboring nodal or distant BLBC metastases [53, 54]. While these observations, coupled with early pre-clinical studies of EGFR TKIs, suggested that EGFR would be a targetable in breast cancer, to date there has been minimal effect of TKIs in clinical trials, even when patient are stratified for EGFR overexpression [55–57]. One potential explanation is that certain EGFR mutations, which predict TKI sensitivity in lung cancer, are rare in breast cancer [58, 59]. However, even rare breast cancers harboring these specific EGFR mutations are not sensitive to TKI treatment [60]. Thus, there is a need to identify alternate resistance mechanisms to EGFR TKIs in breast cancer. In the forward genetics screen, Lee *et al*. utilized an elegant phenotypic reversion assay in 3D laminin-rich gel. In their screen, malignant HMECs, which grow as depolarized, disorganized, proliferative colonies in laminin-rich basement membrane (lrBM), can be reverted to a non-malignant phenotype (non-proliferative, acinus-like structures with proper basal polarity) by EGFR TKIs [25] (Figure 2). Expression of cDNAs that prevent TKI activity (such as FAM83A) allow cells to maintain their malignant phenotype even in the presence of EGFR TKIs [25]. Additional studies confirmed FAM83A also conferred resistance to lapatinib, another EGFR TKI that also targets HER2 [25].

Analysis of human breast cancer samples *via* immunohistochemistry and a real-time PCR array revealed that FAM83A is overexpressed in breast cancer when compared to normal breast [25]. Furthermore, patients with increased FAM83A expression have significantly poorer clinical outcomes [25]. The genomic location of FAM83A is also of interest. The 8q24 region is known to contain the oncogene Myc, which is frequently amplified in cancer [61]. Although FAM83A is also often amplified, not all cases of FAM83A overexpression found in breast cancer result from amplification, indicating that FAM83A overexpression is not merely a bystander in 8q24 amplification [25]. Exogenous expression of FAM83A resulted in increased growth, colony formation in soft agar, and invasion *in vitro*, as well as increased tumor volume in immunocompromised mice *in vivo*. Depletion of FAM83A by shRNA in malignant HMT3522 T4-2 HMECs and the breast cancer cell line MDA-MB-468 led to reduced invasiveness, proliferation rate, clonogenic potential, and tumor volume in immunocompromised mice. Further investigation revealed a role for FAM83A downstream of EGFR in the MAPK and PI3K/AKT signaling cascades [25]. FAM83A-overexpressing HMT3522 T4-2 cells are resistant to both EGFR and PI3K inhibitors, but not to MEK inhibitors, suggesting that FAM83A lies downstream of EGFR and PI3K, but upstream of MEK. shRNA-mediated knockdown of FAM83A in HMT3522 T4-2 and MDA-MB-468 cells resulted in decreased p-ERK and p-AKT, indicating the necessity of FAM83A in the MAPK and PI3K/AKT pathways. Co-immunoprecipitation experiments revealed that FAM83A interacts with both c-Raf and the p85 regulatory subunit of PI3K [25]. These endogenous interactions, as well as tyrosine phosphorylation of FAM83A, increase following stimulation with EGF. Taken together, these findings suggest that FAM83A can be phosphorylated upon EGFR activation, and that phosphorylation may be important for FAM83A-mediated signaling complex formation. Additional work will be needed to identify how FAM83A phosphorylation may regulate signaling complex formation and FAM83A-mediated transformation.

In addition to its role in TKI resistance, FAM83A may also confer resistance to trastuzumab, a monoclonal antibody used in the treatment of HER2+ breast cancer. Trastuzumab directly targets the HER2 (ErbB2) receptor by binding to domain IV in the extracellular region. The drug functions through several mechanisms that include blockade of downstream signaling and prevention of HER2 cleavage into a catalytically active cytoplasmic fragment [62]. Although the introduction of trastuzumab resulted in improved patient outcomes, like erlotinib, acquired and *de novo* resistance remain a major problem [63]. Boyer *et al.* identified FAM83A as one of the most highly tyrosine-phosphorylated proteins in trastuzumab-resistant breast cancer cells when compared to isogenic, trastuzumab-sensitive cells [27]. This finding is consistent with the idea that FAM83A serves as a key intermediate in ErbB-family signaling that is potentially phosphorylated by active ErbB receptors, and again hints that FAM83 phosphorylation status is an intriguing area of future study. Importantly, siRNA-mediated knockdown of FAM83A mRNA expression re-sensitized these cells to trastuzumab, indicating the necessity of FAM83A for the resistant phenotype [27]. Thus, inhibition of FAM83A could re-sensitize breast cancers to multiple precision therapies, including EGFR TKIs and trastuzumab. Designing efficient methods to suppress the functions of FAM83A may have significant therapeutic value.

## A CLOSER LOOK AT FAM83B

Simultaneous to the discovery of FAM83A as a driver of TKI resistance, the Jackson laboratory discovered FAM83B as a driver of human mammary epithelial cell (HMEC) transformation [22]. Using a Validation-Based Insertional Mutagenesis (VBIM) strategy to identify genes that promote the anchorage-independent growth of non-transformed HMEC, the Jackson laboratory found that retroviral insertion into the FAM83B gene, which elevated FAM83B expression, was sufficient to promote HMEC transformation similar to mutant RAS [22] (Figure 2). The discovery of FAM83B gained significance when we noted that a number of human cancers, including breast, bladder, testis, ovary, thyroid, and lung cancers exhibited elevated expression of FAM83B (Table 1) [22]. Interestingly, elevated FAM83B expression is associated with estrogen-receptor (ER) and progesterone-receptor (PR) negative breast tumors, an aggressive subtype for which currently no targeted therapies exist [22, 64]. In addition, FAM83B mRNA expression is significantly higher in lung squamous cell carcinoma when compared to normal adjacent tissue or adenocarcinoma [24, 65]. This finding led to the recommendation that FAM83B is a biomarker and a prognostic marker, as high expression of FAM83B correlates with poor disease-free survival of patients with lung squamous cell carcinoma [65]. In addition to gene amplification and mRNA upregulation, evidence from publically available databases indicates mutations in FAM83B in 12-21% of melanomas [66, 67]. While intriguing, all studies thus far have examined FAM83B overexpression; the function of reported FAM83B mutations is not clear.

FAM83B interacts with a noteworthy list of signaling proteins that facilitate its role as a signaling oncoprotein. For example, the Jackson laboratory has shown that FAM83B blocks the inhibitory interaction of 14-3-3 proteins with CRAF, promoting CRAF membrane localization and thereby inducing activation of MAPK signaling pathway and supporting tumor growth [22]. The Jackson laboratory also established an interaction between FAM83B and EGFR that was required for increased EGFR and phospholipase D1 (PLD1) activation [68]. PLD activity results in the production of phosphatidic acid (PA), a signaling lipid involved in activating mTOR, thus explaining how elevated FAM83B expression drives the activation of PI3K/AKT/mTOR signaling. Additionally, the binding of FAM83B to AKT and the p85 and p110 subunits of PI3K further promotes PI3K/AKT activation [23], although the mechanism of how FAM83B interactions result in signaling activation is not yet clear. Akin to FAM83A, the ability of FAM83B to promote aberrant signaling also correlates with resistance of breast cancer cells to EGFR TKI therapies [22]. Moreover, FAM83B-transformed cells are less sensitive to PI3K, AKT and mTOR targeted therapies [23] (Figure 3). These findings suggest that the level of FAM83B may be an important factor to consider when determining which patient receives these precision medicines in the future.

The involvement of FAM83B in multiple signaling pathways strengthens its potential for precision therapeutic targeting. As mentioned, precision medicines have targeted the PI3K/AKT/mTOR and MEK/ERK signaling pathways. However, selective inhibition of MEK/ERK signaling has consistently induced compensatory activation of PI3K/AKT signaling and vice versa, ultimately conferring resistance. The sensible option would be to simultaneously target MAPK and PI3K/AKT/mTOR signaling, hoping to dampen the compensatory signaling. This approach continues to suffer from the added complexity of harming normal tissues that rely on these pathways. Given that FAM83B overexpression can induce multiple EGFR signaling pathways including EGFR/PLD, MAPK, PI3K/AKT/mTOR, we hypothesized that inhibition of FAM83B might suppress multiple pathways and prevent the compensatory survival signaling. Indeed, ablation of FAM83B in breast and colon cancer cells significantly suppressed tumor cell growth and *in vivo* tumorigenicity [22]. The growth suppression upon FAM83B ablation was coupled with decreased CRAF, PI3K, and AKT membrane localization and a subsequent suppression of activating phosphorylation of both ERK1/2 and AKT [22].

Not only does FAM83B activate MAPK and PI3K/AKT/mTOR signaling, but it may also participate in cancer-associated Wnt signaling. In a proteomics effort to identify important players in the Wnt signaling pathway, FAM83B was identified as a novel interacting partner of negative regulatory proteins Axin-1 and APC [69]. While such interactions are intriguing, the role of FAM83B in regulating Wnt signaling has not been described. These additional FAM83B interactions however, reinforce its importance in cancer cell signaling and suggest the exciting possibility that therapeutically targeting FAM83B may inhibit multiple signaling pathways simultaneously.

## OTHER FAM83 PROTEINS

Gene expression studies reveal that FAM83 members are not only elevated in a wide variety of cancers, but in many instances more than one of the FAM83 proteins are significantly elevated. For example, the Jackson laboratory recently described that FAM83A, B, D and E are all highly expressed in ovarian cancers, and similarly bladder cancers show elevated levels of FAM83A, C and D [24]. While the reason a cancer cell upregulates multiple FAM83 proteins is unclear, we propose that each FAM83 protein likely has both redundant and non-redundant roles in oncogenic signaling. Until recently, not much was known about the other six members (FAM83C-H) of the FAM83 family in the context of cancer induction or maintenance. Although initially annotated as “hypothetical proteins”, the expression of each FAM83 protein has now been confirmed by mass spectrometry [24]. FAM83 proteins share the conserved amino-terminal domain of unknown function (DUF1669), but this domain has no characterized function. The only enzymatic activity predicted in the FAM83 proteins was based on a PLD-like motif within the DUF1669, although when tested, neither FAM83A nor FAM83B possessed conventional PLD activity [22, 25]. However, the DUF1669 domain in FAM83B is necessary and sufficient for EGFR and CRAF binding and FAM83B-mediated transformation [22, 68]. In addition, transformation-inducing FAM83A is only 434 amino acids and is comprised mainly of the DUF1669. Therefore, it is conceivable that all FAM83 proteins contribute to transformation by way of the DUF1669, similar to FAM83A and FAM83B. Indeed, recent observations confirm that FAM83 members A-E can independently promote anchorage-independent growth, and form complexes with CRAF in mammary epithelial cells [24]. Moreover, Wang *et al.* recently linked elevated expression of FAM83D and its ability to promote MEK/ERK signaling with higher incidence of hepatocellular carcinomas (HCC) [70, 71].

In addition to redundant transformative properties, FAM83 proteins likely also have non-redundant functions. These proteins lack any significant homology beyond the DUF1669 and vary greatly in size ranging from 434-1179 amino acids (Figure 1). FAM83G, for example, may participate in EGFR signaling by interacting with regulator for ubiquitous kinase/c-Cbl interacting protein of 85KDa (Ruk/CIN85) [72]. Ruk/CIN85 is known to promote invasiveness and migration of breast cancer cells *via* regulation of EGF-driven Src/AKT signaling pathways [73]. FAM83G is also implicated in TGF-ß signaling as mass spectrometry analysis identified it as a novel interacting partner of Smad2 and Smad3 [74]. Recently, FAM83G was reported as a substrate of type I Bone Morphogenic Protein (BMP) receptor whereby it associates with Smad1 and promotes non-canonical Smad4-independent signaling and transcription [75]. Of note, dysregulated TGF-ß and BMP signaling is associated with cancer development and recurrence [76]. Moreover, FAM83G also induced gene transcription of additional non-BMP target genes clearly pointing to a wider role in cellular processes.

FAM83D (originally referred to as CHICA) binds to the chromokinesin KID and localizes to the spindle during mitosis to regulate spindle maintenance and cell division [77]. Accordingly, a meta-analysis of FAM83D indicated that FAM83D is co-expressed with key genes related to mitotic-progression and cytokinesis [78]. The FAM83D/KID interaction occurs independently of the FAM83D DUF1669, relying on the C-terminus of FAM83D, which has no significant similarity with other FAM83 proteins [77]. FAM83D also interacts with F-box/WD repeat-containing protein 7 (FBXW7) resulting in the down-regulation of FBXW7, a suppressor of c-Myc, mTOR, and C-Jun expression [79]. Thus, elevated expression of FAM83D increases the expression of these downstream oncogenes, which likely contributes to its ability to drive transformation. Indeed, as observed with FAM83A and FAM83B, the gene amplification and elevated protein expression of FAM83D increased the migration and invasion of breast epithelial cells, and was associated with poor prognosis [79]. As FAM83D regulates tumorigenesis by hyperactivating mTOR, the levels of FAM83D may also predict patient response to rapamycin [79]. While the regulation of most FAM83 proteins is poorly understood, FAM83D expression is tightly regulated. FAM83D is post-translationally regulated by type I Arginine methyltransferase PRMT-1 that targets FAM83D for arginine methylation and likely enhances protein turnover or promotes protein-protein interactions [80]. FAM83D transcription is also regulated by miR-210, which is induced by hypoxia and functions as a tumor suppressor by inhibiting the expression of multiple genes involved in promoting cell division, including FAM83D [81]. Similar to FAM83D, FAM83F expression is regulated by a microRNA, miR-143, that degrades the FAM83F mRNA transcript to exert a tumor-suppressive effect in esophageal squamous cell carcinoma cells [82].

Similar to the previously identified role of FAM83A and FAM83B in resistance to EGFR TKI inhibitors, newer reports implicate FAM83H in therapeutic resistance. An insertional mutagenesis screen to find genes that confer Erlotinib resistance in pancreatic cancers identified a common integration site, LOC100128338, which shares chromosomal location with FAM83H [83]. Interestingly, a similar screen in an orthotopic mouse model identified FAM83H as one of 11 genes that promote androgen-independent prostate cancer [84]. Therefore, FAM83H appears to be an important player in erlotinib-resistant pancreatic cancer and castration-resistant prostate cancer, both of which are extremely difficult to treat. Unrelated to cancer, a separate series of reports have implicated mutations in FAM83H as the cause of hypocalcified Amelogenesis imperfecta (AI), a hereditary condition in which the enamel of the teeth does not mineralize to the level of normal enamel [85–87]. A FAM83HUnexpectedly, the FAM83H knockout mouse had no tooth malformations or defects in enamel formation. However, the authors did report an association between FAM83H and Casein Kinase Iε. This interaction facilitates the cytoplasmic localization of Casein Kinase Iε and the C-terminal phosphorylation of FAM83H to create docking sites for other proteins. The authors propose that the FAM83H mutations linked to AI are likely gain of function mutations that do not interfere with normal FAM83H functions, but lead to mislocalized nuclear Casein Kinase Iε that is toxic to ameloblasts. Interestingly, of the numerous inherited FAM83H mutations noted as the cause of AI, there appears to be no increase in susceptibility to cancer. In addition interacting with casein kinases in development, FAM83H interacts with a casein-kinase isoform that has a role in colon cancer. A DUF1669-independent association between FAM83H, keratin 18, and casein kinase −1α (CK-1α) results in CK-1-mediated disassembly of keratin filaments and a loss of epithelial cell polarity, which may have significant implications in colon cancer invasion and metastasis [89]. Although DUF1669-independent functions have not yet been identified for other FAM83 members, the evidence with FAM83D and FAM83H opens up exciting new possibilities for the cellular roles of FAM83 proteins. It is thought-provoking to explore the divergent C-terminal sequences of FAM83 proteins so as to understand their unique, non-redundant functions. Future work should include detailed *in silico*, bioinformatics analysis on the C-terminal sequences in combination with cellular assays using C-terminal swap/deletion mutants of FAM83 proteins.

## PROPOSED MECHANISM OF FAM83 ACTION

While several FAM83 proteins have been implicated in receptor-mediated signaling pathways (EGFR, TGFβ, BMP) and in modulating their downstream effectors, the mechanism by which elevated FAM83 expression hyperactivates each of these pathways remains unclear. Moreover, given the potential redundant and non-redundant functions described in the previous section, the manner in which each FAM83 protein contributes to oncogenic signaling may be unique. Here, we discuss potential modes of action for the FAM83 proteins.

Evidence to date supports an adaptor/scaffold function for the FAM83 proteins since they lack obvious enzymatic functions, yet interact with essential signaling intermediaries. Current known FAM83 interacting proteins include EGFR, RAF, PI3K-p85, PI3K-p110, AKT, Axin-1, and APC [22–25, 68, 69]. Moreover, other interactions are noted in the Biogrid interaction repository between FAM83 proteins and key cancer-associated proteins such as BRCA1 (FAM83A), CK1α, ß, and δ (FAM83D, G, and H), GADD45 (FAM83D), GSK3ß (FAM83D), RHAMM (FAM83D), CD10 (FAM83F), Human Src Family Kinase-Binding Protein 1 (FAM83G), SMAD2 and SMAD3 (FAM83G), FBXW11 (FAM83H), SKP2 (FAM83H) [90]. Phosphorylation of FAM83 proteins also supports their function as adaptor proteins. In large phospho-proteomic screens, multiple kinases have been implicated in the phosphorylation and potential regulation of FAM83 proteins, including EGFR, PDK, AKT, CDKs, MAPK, SRC, RSK, GSK3b, PKA, PKC, and PLK [91]. Phosphorylation plays a vital role in intracellular signal transduction, often by initiating a structural change in the phosphorylated protein that facilitates the recruitment of additional signaling partners. Likewise, aberrant phosphorylation of signaling effectors in cancer cells often drives unchecked growth and proliferation. We predict that the increased presence of hyper-phosphorylated FAM83 proteins may nucleate, and thus hyperactivate, oncogenic signaling cascades.

In addition to kinase signaling, FAM83 proteins may have additional adaptor functions in cancer cells, including nucleating and inactivating enzymes that destabilize oncogenic signaling proteins [30]. FAM83D has recently been shown to interact with the E3 ubiquitin ligase, FBXW7 and inhibit the degradation of mTOR, MYC, and JUN [79]. FBXW7 is one of the subunits of the SCF (SKP1-cullin-FBXW7) ubiquitin ligase complex, which functions in phosphorylation-dependent ubiquitination [92]. In fact, FAM83D has a predicted FBXW7 degradation sequence [93], yet interaction between FAM83D and FBXW7 does not lead to FAM83D degradation. Rather, the FAM83D/ FBXW7 interaction results in inhibition of FBXW7 ubiquitin ligase activity [79]. This finding brings up the interesting possibility that other FAM83 proteins may also suppress FBXW7 ubiquitin ligase activity, and potentially other ubiquitin ligases. In fact, FAM83 proteins contain a predicted destruction D-box motif (all FAM83 proteins) and KEN-box motif (FAM83B) for targeting by the anaphase-promoting complex (APC/C) ubiquitin ligase [93]. Thus, FAM83 proteins may inhibit E3 ubiquitin ligases, leading to accumulation of various downstream substrates with known roles in carcinogenesis [94]. The numerous potential interactions provide avenues for future study into the redundant and non-redundant functions of the FAM83 proteins in oncogenesis, and underscore the complexity of this novel oncogene family.

## THERAPEUTIC PROSPECTS AND CHALLENGES

**Figure 4:**
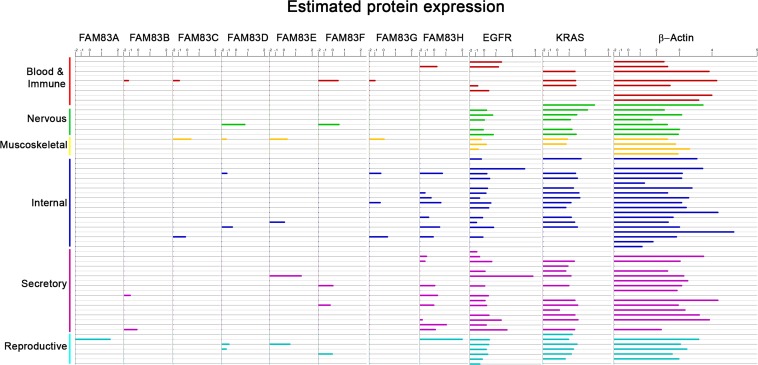
Normal Tissue Expression Levels of FAM83 Analysis of the human proteome across most major tissue types showing expression of FAM83A-H members and oncogenes EGFR and RAS alongside beta-actin. The tissue types include the following: Blood and Immune (Serum, Plasma, Monocyte, Peripheral Blood mononuclear cells, Platelet, Lymph node, Tonsil, Bone marrow stromal cell, Bone marrow mesenchymal stem cell), Nervous (Brain, Fetal Brain, Frontal cortex, Cerebral cortex, Cerebrospinal fluid, Spinal Cord, Retina), Muscoskeletal (Heart, Fetal Heart, Bone, Colon muscle), Internal (Oral epithelium, Nasopharynx, Nasal respiratory epithelium, Esophagus, Stomach, Cardia, Fetal gut, Colon, Rectum, Liver, Fetal liver, Liver secretome, Kidney, Spleen, Lung, Lung Alveolar lavage, Adipocyte, Synovial fluid, Amniocyte), Secretory (Vitreous humor, Saliva, Salivary gland, Thyroid, Adrenal, Breast, Milk, Pancreas, Pancreatic Juice, Islet of Langerhans, Gallbladder, Prostate, Urine, Urinary Bladder, Skin, Hair Follicle, Placenta), Reproductive (Uterus, Cervix, Ovary, Fetal Ovary, Testis, Fetal Testis, Seminal vesicle).

Analysis of the human proteome across most major tissue types indicates that FAM83 members are expressed at low (often undetectable) levels throughout the body relative to other proto-oncogenes such as EGFR and RAS (Figure 4). When coupled with the observed overexpression of FAM83 members in diverse cancers, we propose that the potentially large therapeutic window may provide new methods to target EGFR/RAS-driven tumors. However, significant challenges remain in determining how to best target the oncogenic functions of the FAM83 members. To date, published findings have largely been reliant on overexpression and RNAi approaches using non-transformed and cancerous cell lines, respectively. Knowledge about how the FAM83 proteins function in normal biology and normal tissue homeostasis is currently lacking for nearly all FAM83 members, because mouse models have not yet been generated. Currently, only one FAM83 knockout animal (FAM83H) exists, which, while viable, dies within two weeks after birth [88]. The postnatal lethality of mice lacking FAM83H suggests that FAM83H is not required for normal development, but rather may be required for cellular or tissue homeostasis. The development of additional knock-out or transgenic models lacking or expressing elevated levels of the FAM83 proteins will be important future studies. Such models will provide functional genetic evidence of each FAM83 member's importance, or lack of importance, in normal development and homeostasis.

A greater understanding of the mechanisms by which the FAM83 proteins enhance and prolong oncogenic signaling will also be critical for determining whether and how each FAM83 protein can be targeted. Studies have shown FAM83 proteins interact with many key cancer-signaling proteins including EGFR (FAM83B), PI3K (FAM83A-B), AKT (FAM83B), c-RAF (FAM83A-E) [22–25, 68]. Scansite motif scan identifies numerous potential SH2-binding (phosphotyrosine residues) and SH3-binding (proline-rich sequences) motifs on each FAM83 protein, and each is extensively phosphorylated [95]. Thus, based on the available data, we hypothesize that FAM83 members function to direct the assembly or enhance the stability of oncogenic signaling complexes. Targeting the ability of FAM83 proteins to assemble or stabilize signaling complexes may suppress effector activation, but unlike enzymes, which can be targeted by blocking catalytic activity, inhibiting adaptor protein function requires a tailored approach. Once considered “undruggable”, there now exist numerous examples of drugs that target protein interfaces to block protein-protein interactions. These include p53/MDM2, β-Catenin/CREB, IAP/SMAC, tubulin-α/tubulin β, BCL2/BAX, BCL2/BAK, mTOR/FKBP12, and BCL2/Beclin, and components of the MAPK and PI3K pathways, where FAM83 members are also known to interact [96]. IQGAP1 is a known MAPK scaffold that binds ERK1/2, MEK1/2, and RAF [97, 98]. Exogenous treatment with a “WW peptide,” which mimics the ERK-binding region of IQGAP1, inhibits IQGAP1/ERK binding and increased survival in murine models of RAS-driven cancers [99]. Moreover, a mutant PI3K (p110α) peptide capable of disrupting p110α/IRS1 complexes inhibits mutant p110α-activity and suppresses tumorigenicity [100]. Small molecule inhibitors have also been developed to disrupt MAPK pathway adaptors. Prohibitin works to assemble C-Raf and RAS at the plasma membrane in RAS-driven pancreatic cancer cells [101]. Rocaglamide, a small molecule inhibitor that binds to prohibitin, blocks prohibitin/c-Raf interactions and suppresses downstream signaling [102]. Additionally, a small molecule RAS-mimetic, rigosertib was recently described to efficiently block the RAS-binding domain on RAF successfully inhibiting the oncogenic RAS-RAF-MEK pathway [103].

Thus, while feasible, rational drug design and *in silico* attempts at disrupting adaptor interactions depend on sufficient knowledge of the protein structure and protein-binding interfaces, which are currently lacking for most FAM83 proteins. The only crystal structure solved is of FAM83A, which is the smallest FAM83 protein, largely consisting of the DUF1669 domain [104]. Again, all FAM83 members share this domain, and studies indicate that protein interactions mediated by DUF1669 are important for driving transformation [22, 68]. For example, mutating K230 within the DUF1669 of FAM83B, an amino acid highly conserved among FAM83 members, weakens the FAM83B/EGFR interaction and suppresses downstream effector activation [68]. We propose that the right small molecule or peptide could function similarly to suppress critical protein interactions necessary for the oncogenic functions of the FAM83 proteins. Defining which FAM83 interactions to target, however, is a challenging task, especially given the potential redundant and non-redundant functions of the 8 FAM83 members.

Another method for targeting adaptor proteins is to suppress their expression using RNA interference (RNAi). RNA interference (RNAi) as a therapeutic regimen is continuously improving and holds great potential [105–108]. siRNA encapsulated nanoparticles targeting oncogenic mRNA transcripts such as VEGF, KRASG12D, and EphA2 are currently in clinical trials for solid tumors [109]. Recent developments in cationic-lipid-based siRNA carriers have been described to efficiently deliver the siRNA cargo intracellularly. When coupled to a targeting peptide such as Arg-Gly-Asp (RGD), which is recognized by β3 integrin in the tumor vasculature, nanoparticles can efficiently target the delivery of siRNA to the tumor [110]. Such particles can now be injected systemically *in vivo*, with little immunogenicity, no changes in body weight, and no issues with clotting [110]. The rapid and continuing advancements in RNAi strategies will make the targeting of FAM83 members a more feasible option in the near term.

## CONCLUSION AND FUTURE DIRECTIONS

The significant discovery of a new family of oncogenes as critical mediators of EGFR/RAS signaling provides the basis for the development of novel therapeutics that target FAM83 members in a wide variety of cancers. The tumor-exclusive increase in expression of FAM83 proteins makes them highly desirable targets. It is also clear that sensitivity to select targeted therapies is closely linked to the expression of the FAM83 oncogenes, and screening for FAM83 expression will help predict response to treatment strategies as well as to determine future therapeutic options. We envision future studies will involve identification of the unique function of each FAM83 protein, and development of new strategies to therapeutically target both the conserved and unique oncogenic properties of FAM83 proteins.

## References

[1] Hanahan D, Weinberg Robert A (2011). Hallmarks of Cancer: The Next Generation. Cell.

[2] Sherr CJ (2004). Principles of Tumor Suppression. Cell.

[3] Hanahan D, Weinberg RA (2000). The Hallmarks of Cancer. Cell.

[4] Blum G, Gazit A, Levitzki A (2000). Substrate Competitive Inhibitors of IGF-1 Receptor Kinase. Biochemistry.

[5] Burotto M, Manasanch EE, Wilkerson J, Fojo T (2015). Gefitinib and Erlotinib in Metastatic Non-Small Cell Lung Cancer: A Meta-Analysis of Toxicity and Efficacy of Randomized Clinical Trials. The Oncologist.

[6] Ciardiello F, Caputo R, Bianco R, Damiano V, Pomatico G, De Placido S, Bianco AR, Tortora G (2000). Antitumor Effect and Potentiation of Cytotoxic Drugs Activity in Human Cancer Cells by ZD-1839 (Iressa), an Epidermal Growth Factor Receptor-selective Tyrosine Kinase Inhibitor. Clinical Cancer Research.

[7] Cox AD, Fesik SW, Kimmelman AC, Luo J, Der CJ (2014). Drugging the undruggable Ras: mission possible?. Nature reviews Drug discovery.

[8] Davis NM, Sokolosky M, Stadelman K, Abrams SL, Libra M, Candido S, Nicoletti F, Polesel J, Maestro R, D'Assoro A, Drobot L, Rakus D, Gizak A, Laidler P, Duliska-Litewka J, Basecke J (2014). Deregulation of the EGFR/PI3K/PTEN/Akt/mTORC1 pathway in breast cancer: possibilities for therapeutic intervention. Oncotarget.

[9] Lennartsson J, Rönnstrand L (2012). Stem Cell Factor Receptor/c-Kit: From Basic Science to Clinical Implications. Physiological Reviews.

[10] Martin-Liberal J, Larkin J (2014). New RAF kinase inhibitors in cancer therapy. Expert Opinion on Pharmacotherapy.

[11] Parikh RA, Wang P, Beumer JH, Chu E, Appleman LJ (2014). The potential roles of hepatocyte growth factor (HGF)-MET pathway inhibitors in cancer treatment. OncoTargets and therapy.

[12] Roskoski Jr R (2015). A historical overview of protein kinases and their targeted small molecule inhibitors. Pharmacological Research.

[13] Sun S-Y (2013). mTOR kinase inhibitors as potential cancer therapeutic drugs. Cancer letters.

[14] Vogel CL, Cobleigh MA, Tripathy D, Gutheil JC, Harris LN, Fehrenbacher L, Slamon DJ, Murphy M, Novotny WF, Burchmore M, Shak S, Stewart SJ, Press M (2002). Efficacy and Safety of Trastuzumab as a Single Agent in First-Line Treatment of HER2-Overexpressing Metastatic Breast Cancer. Journal of Clinical Oncology.

[15] Yap TA, Bjerke L, Clarke PA, Workman P (2015). Drugging PI3K in cancer: refining targets and therapeutic strategies. Current Opinion in Pharmacology.

[16] Dienstmann R, Rodon J, Prat A, Perez-Garcia J, Adamo B, Felip E, Cortes J, Iafrate AJ, Nuciforo P, Tabernero J (2014). Genomic aberrations in the FGFR pathway: opportunities for targeted therapies in solid tumors. Annals of Oncology.

[17] Holohan C, Van Schaeybroeck S, Longley DB, Johnston PG (2013). Cancer drug resistance: an evolving paradigm. Nat Rev Cancer.

[18] Logue JS, Morrison DK (2012). Complexity in the signaling network: insights from the use of targeted inhibitors in cancer therapy. Genes & Development.

[19] Mancini M, Yarden Y (2016). Mutational and Network Level Mechanisms Underlying Resistance to Anti-cancer Kinase Inhibitors. Seminars in Cell & Developmental Biology.

[20] McCubrey JA, Steelman LS, Chappell WH, Abrams SL, Franklin RA, Montalto G, Cervello M, Libra M, Candido S, Malaponte G, Mazzarino MC, Fagone P, Nicoletti F, Bäsecke J, Mijatovic S, Maksimovic-Ivanic D (2012). Ras/Raf/MEK/ERK and PI3K/PTEN/Akt/mTOR Cascade Inhibitors: How Mutations Can Result in Therapy Resistance and How to Overcome Resistance. Oncotarget.

[21] Rozengurt E, Soares HP, Sinnet-Smith J (2014). Suppression of feedback loops mediated by PI3K/mTOR induces multiple over-activation of compensatory pathways: an unintended consequence leading to drug resistance. Molecular cancer therapeutics.

[22] Cipriano R, Graham J, Miskimen KLS, Bryson BL, Bruntz RC, Scott SA, Brown HA, Stark GR, Jackson MW (2012). FAM83B mediates EGFR- and RAS-driven oncogenic transformation. The Journal of Clinical Investigation.

[23] Cipriano R, Miskimen KLS, Bryson BL, Foy CR, Bartel CA, Jackson MW (2013). FAM83B-mediated activation of PI3K/AKT and MAPK signaling cooperates to promote epithelial cell transformation and resistance to targeted therapies. Oncotarget.

[24] Cipriano R, Miskimen KLS, Bryson BL, Foy CR, Bartel CA, Jackson MW (2014). Conserved Oncogenic Behavior of the FAM83 Family Regulates MAPK Signaling in Human Cancer. Molecular cancer research : MCR.

[25] Lee S-Y, Meier R, Furuta S, Lenburg ME, Kenny PA, Xu R, Bissell MJ (2012). FAM83A confers EGFR-TKI resistance in breast cancer cells and in mice. The Journal of Clinical Investigation.

[26] Yates A, Akanni W, Amode MR, Barrell D, Billis K, Carvalho-Silva D, Cummins C, Clapham P, Fitzgerald S, Gil L, Girón CG, Gordon L, Hourlier T, Hunt SE, Janacek SH, Johnson N (2016). Ensembl 2016. Nucleic Acids Research.

[27] Boyer AP, Collier TS, Vidavsky I, Bose R (2013). Quantitative Proteomics with siRNA Screening Identifies Novel Mechanisms of Trastuzumab Resistance in HER2 Amplified Breast Cancers. Molecular & Cellular Proteomics : MCP.

[28] Yarden Y, Sliwkowski MX (2001). Untangling the ErbB signalling network. Nat Rev Mol Cell Biol.

[29] Yarden Y (2001). The EGFR family and its ligands in human cancer: signalling mechanisms and therapeutic opportunities. European Journal of Cancer.

[30] Avraham R, Yarden Y (2011). Feedback regulation of EGFR signalling: decision making by early and delayed loops. Nat Rev Mol Cell Biol.

[31] Henson ES, Gibson SB (2006). Surviving cell death through epidermal growth factor (EGF) signal transduction pathways: Implications for cancer therapy. Cellular Signalling.

[32] Leicht DT, Balan V, Kaplun A, Singh-Gupta V, Kaplun L, Dobson M, Tzivion G (2007). Raf Kinases: Function, Regulation and Role in Human Cancer. Biochimica et biophysica acta.

[33] Klempner SJ, Myers AP, Cantley LC (2013). What a Tangled Web We Weave: Emerging Resistance Mechanisms to Inhibition of the Phosphoinositide 3-kinase Pathway. Cancer discovery.

[34] Xu K, Liu P, Wei W (2014). mTOR signaling in tumorigenesis. Biochimica et Biophysica Acta (BBA) - Reviews on Cancer.

[35] Yarden Y, Pines G (2012). The ERBB network: at last, cancer therapy meets systems biology. Nat Rev Cancer.

[36] Ostrem JM, Peters U, Sos ML, Wells JA, Shokat KM (2013). K-Ras(G12C) inhibitors allosterically control GTP affinity and effector interactions. Nature.

[37] Girotti Maria R, Lopes F, Preece N, Niculescu-Duvaz D, Zambon A, Davies L, Whittaker S, Saturno G, Viros A, Pedersen M, Suijkerbuijk Bart M, Menard D, McLeary R, Johnson L, Fish L, Ejiama S (2015). Paradox-Breaking RAF Inhibitors that Also Target SRC Are Effective in Drug-Resistant BRAF Mutant Melanoma. Cancer Cell.

[38] Holderfield M, Deuker MM, McCormick F, McMahon M (2014). Targeting RAF kinases for cancer therapy: BRAF mutated melanoma and beyond. Nature reviews Cancer.

[39] Zhao Y, Adjei AA (2014). The clinical development of MEK inhibitors. Nat Rev Clin Oncol.

[40] Dienstmann R, Rodon J, Serra V, Tabernero J (2014). Picking the Point of Inhibition: A Comparative Review of PI3K/AKT/mTOR Pathway Inhibitors. Molecular Cancer Therapeutics.

[41] Chappell WH, Steelman LS, Long JM, Kempf RC, Abrams SL, Franklin RA, Bäsecke J, Stivala F, Donia M, Fagone P, Malaponte G, Mazzarino MC, Nicoletti F, Libra M, Maksimovic-Ivanic D, Mijatovic S (2011). Ras/Raf/MEK/ERK and PI3K/PTEN/Akt/mTOR Inhibitors: Rationale and Importance to Inhibiting These Pathways in Human Health. Oncotarget.

[42] Samatar AA, Poulikakos PI (2014). Targeting RAS-ERK signalling in cancer: promises and challenges. Nat Rev Drug Discov.

[43] Russo A, Franchina T, Rita Ricciardi GR, Picone A, Ferraro G, Zanghì M, Toscano G, Giordano A, Adamo V (2015). A decade of EGFR inhibition in EGFR-mutated non small cell lung cancer (NSCLC): Old successes and future perspectives. Oncotarget.

[44] Groenendijk FH, Bernards R (2014). Drug resistance to targeted therapies: Déjà vu all over again. Molecular Oncology.

[45] Fruman DA, Rommel C (2014). PI3K and cancer: lessons, challenges and opportunities. Nat Rev Drug Discov.

[46] Li Y, Dong X, Yin Y, Su Y, Xu Q, Zhang Y, Pang X, Zhang Y, Chen W (2005). BJ-TSA-9, a Novel Human Tumor-Specific Gene, Has Potential as a Biomarker of Lung Cancer. Neoplasia (New York, NY).

[47] Liu LEI, Ma C, Xu Q, Cheng L, Xiao L, Xu D, Gao Y, Wang J, Song H (2014). A rapid nested polymerase chain reaction method to detect circulating cancer cells in breast cancer patients using multiple marker genes. Oncology Letters.

[48] Citri A, Yarden Y (2006). EGF–ERBB signalling: towards the systems level. Nat Rev Mol Cell Biol.

[49] Chong CR, Jänne PA (2013). The quest to overcome resistance to EGFR-targeted therapies in cancer. Nature medicine.

[50] Dienstmann R, Rodon J, Serra V, Tabernero J (2011). Personalizing Therapy with Targeted Agents in Non-Small Cell Lung Cancer. Oncotarget.

[51] Roberts PJ, Der CJ (2007). Targeting the Raf-MEK-ERK mitogen-activated protein kinase cascade for the treatment of cancer. Oncogene.

[52] Hoadley KA, Weigman VJ, Fan C, Sawyer LR, He X, Troester MA, Sartor CI, Rieger-House T, Bernard PS, Carey LA, Perou CM (2007). EGFR associated expression profiles vary with breast tumor subtype. BMC Genomics.

[53] Nielsen TO, Hsu FD, Jensen K, Cheang M, Karaca G, Hu Z, Hernandez-Boussard T, Livasy C, Cowan D, Dressler L, Akslen LA, Ragaz J, Gown AM, Gilks CB, van de Rijn M, Perou CM (2004). Immunohistochemical and Clinical Characterization of the Basal-Like Subtype of Invasive Breast Carcinoma. Clinical Cancer Research.

[54] Viale G, Rotmensz N, Maisonneuve P, Bottiglieri L, Montagna E, Luini A, Veronesi P, Intra M, Torrisi R, Cardillo A, Campagnoli E, Goldhirsch A, Colleoni M (2009). Invasive ductal carcinoma of the breast with the “triple-negative” phenotype: prognostic implications of EGFR immunoreactivity. Breast Cancer Res Treat.

[55] Baselga J, Gómez P, Greil R, Braga S, Climent MA, Wardley AM, Kaufman B, Stemmer SM, Pêgo A, Chan A, Goeminne J-C, Graas M-P, Kennedy MJ, Ciruelos Gil EM, Schneeweiss A, Zubel A (2013). Randomized Phase II Study of the Anti–Epidermal Growth Factor Receptor Monoclonal Antibody Cetuximab With Cisplatin Versus Cisplatin Alone in Patients With Metastatic Triple-Negative Breast Cancer. Journal of Clinical Oncology.

[56] Carey LA, Rugo HS, Marcom PK, Mayer EL, Esteva FJ, Ma CX, Liu MC, Storniolo AM, Rimawi MF, Forero-Torres A, Wolff AC, Hobday TJ, Ivanova A, Chiu W-K, Ferraro M, Burrows E (2012). TBCRC 001: Randomized Phase II Study of Cetuximab in Combination With Carboplatin in Stage IV Triple-Negative Breast Cancer. Journal of Clinical Oncology.

[57] Palma G, Frasci G, Chirico A, Esposito E, Siani C, Saturnino C, Arra C, Ciliberto G, Giordano A, D'Aiuto M (2015). Triple negative breast cancer: looking for the missing link between biology and treatments. Oncotarget.

[58] Secq V, Villeret J, Fina F, Carmassi M, Carcopino X, Garcia S, Metellus I, Boubli L, Iovanna J, Charpin C (2014). Triple negative breast carcinoma EGFR amplification is not associated with EGFR, Kras or ALK mutations. British Journal of Cancer.

[59] Tilch E, Seidens T, Cocciardi S, Reid LE, Byrne D, Simpson PT, Vargas AC, Cummings MC, Fox SB, Lakhani SR, Chenevix Trench G (2014). Mutations in EGFR, BRAF and RAS are rare in triple-negative and basal-like breast cancers from Caucasian women. Breast Cancer Res Treat.

[60] Generali D, Leek R, Fox S, Moore J, Taylor C, Chambers P, Harris A (2007). EGFR mutations in exons 18–21 in sporadic breast cancer. Annals of Oncology.

[61] Tansey WP (2014). Mammalian MYC Proteins and Cancer. New Journal of Science.

[62] Nahta R, Esteva FJ (2007). Trastuzumab: triumphs and tribulations. Oncogene.

[63] D'Amato V, Raimondo L, Formisano L, Giuliano M, De Placido S, Rosa R, Bianco R (2015). Mechanisms of lapatinib resistance in HER2-driven breast cancer. Cancer Treatment Reviews.

[64] Rhodes DR, Kalyana-Sundaram S, Mahavisno V, Varambally R, Yu J, Briggs BB, Barrette TR, Anstet MJ, Kincead-Beal C, Kulkarni P, Varambally S, Ghosh D, Chinnaiyan AM (2007). Oncomine 3. 0: Genes, Pathways, and Networks in a Collection of 18,000 Cancer Gene Expression Profiles. Neoplasia (New York, NY).

[65] Okabe N, Ezaki J, Yamaura T, Muto S, Osugi JUN, Tamura H, Imai J-I, Ito EMI, Yanagisawa Y, Honma R, Gotoh M, Watanabe S, Waguri S, Suzuki H (2015). FAM83B is a novel biomarker for diagnosis and prognosis of lung squamous cell carcinoma. International Journal of Oncology.

[66] Cerami E, Gao J, Dogrusoz U, Gross BE, Sumer SO, Aksoy BA, Jacobsen A, Byrne CJ, Heuer ML, Larsson E, Antipin Y, Reva B, Goldberg AP, Sander C, Schultz N (2012). The cBio Cancer Genomics Portal: An Open Platform for Exploring Multidimensional Cancer Genomics Data. Cancer Discovery.

[67] Gao J, Aksoy BA, Dogrusoz U, Dresdner G, Gross B, Sumer SO, Sun Y, Jacobsen A, Sinha R, Larsson E, Cerami E, Sander C, Schultz N (2013). Integrative Analysis of Complex Cancer Genomics and Clinical Profiles Using the cBioPortal. Science signaling.

[68] Cipriano R, Bryson BL, Miskimen KLS, Bartel CA, Hernandez-Sanchez W, Bruntz RC, Scott SA, Lindsley CW, Brown HA, Jackson MW (2014). Hyperactivation of EGFR and downstream effector phospholipase D1 by oncogenic FAM83B. Oncogene.

[69] Hilger M, Mann M (2012). Triple SILAC to Determine Stimulus Specific Interactions in the Wnt Pathway. Journal of Proteome Research.

[70] Liao W, Liu W, Liu X, Yuan Q, Ou Y, Qi Y, Huang W, Wang Y, Huang J (2015). Upregulation of FAM83D affects the proliferation and invasion of hepatocellular carcinoma. Oncotarget.

[71] Wang D, Han S, Peng R, Wang X, Yang X-X, Yang R-J, Jiao C-Y, Ding D, Ji G-W, Li X-C (2015). FAM83D activates the MEK/ERK signaling pathway and promotes cell proliferation in hepatocellular carcinoma. Biochemical and Biophysical Research Communications.

[72] Havrylov S, Rzhepetskyy Y, Malinowska A, Drobot L, Redowicz MJ (2009). Proteins recruited by SH3 domains of Ruk/CIN85 adaptor identified by LC-MS/MS. Proteome Science.

[73] Samoylenko A, Vynnytska-Myronovska B, Byts N, Kozlova N, Basaraba O, Pasichnyk G, Palyvoda K, Bobak Y, Barska M, Mayevska O, Rzhepetsky Y, Shuvayeva H, Lyzogubov V, Usenko V, Savran V, Volodko N (2012). Increased levels of the HER1 adaptor protein Rukl/CIN85 contribute to breast cancer malignancy. Carcinogenesis.

[74] Brown KA, Ham A-JL, Clark CN, Meller N, Law BK, Chytil A, Cheng N, Pietenpol JA, Moses HL (2008). Identification of Novel Smad2 and Smad3 Associated Proteins in Response to TGF-1. Journal of cellular biochemistry.

[75] Vogt J, Dingwell KS, Herhaus L, Gourlay R, Macartney T, Campbell D, Smith JC, Sapkota GP (2014). Protein associated with SMAD1 (PAWS1/FAM83G) is a substrate for type I bone morphogenetic protein receptors and modulates bone morphogenetic protein signalling. Open Biology.

[76] Gonzalez DM, Medici D (2014). Signaling mechanisms of the epithelial-mesenchymal transition. Science signaling.

[77] Santamaria A, Nagel S, Sillje HHW, Nigg EA (2008). The Spindle Protein CHICA Mediates Localization of the Chromokinesin Kid to the Mitotic Spindle. Current Biology.

[78] Varisli L (2012). Meta-analysis of the expression of the mitosis-related gene Fam83D. Oncology Letters.

[79] Wang Z, Liu Y, Zhang P, Zhang W, Wang W, Curr K, Wei G, Mao J-H (2013). FAM83D promotes cell proliferation and motility by downregulating tumor suppressor gene FBXW7. Oncotarget.

[80] Weimann M, Grossmann A, Woodsmith J, Ozkan Z, Birth P, Meierhofer D, Benlasfer N, Valovka T, Timmermann B, Wanker EE, Sauer S, Stelzl U (2013). A Y2H-seq approach defines the human protein methyltransferase interactome. Nat Meth.

[81] He J, Wu J, Xu N, Xie W, Li M, Li J, Jiang Y, Yang BB, Zhang Y (2013). MiR-210 disturbs mitotic progression through regulating a group of mitosis-related genes. Nucleic Acids Research.

[82] Mao Y, Liu J, Zhang D, Li B (2016). miR-143 inhibits tumor progression by targeting FAM83F in esophageal squamous cell carcinoma. Tumor Biology.

[83] Ranzani M, Annunziato S, Calabria A, Brasca S, Benedicenti F, Gallina P, Naldini L, Montini E (2014). Lentiviral Vector-based Insertional Mutagenesis Identifies Genes Involved in the Resistance to Targeted Anticancer Therapies. Mol Ther.

[84] Nalla AK, Williams TF, Collins CP, Rae DT, Trobridge GD (2015). Lentiviral vector-mediated insertional mutagenesis screen identifies genes that influence androgen independent prostate cancer progression and predict clinical outcome. Molecular Carcinogenesis.

[85] Kim J-W, Lee S-K, Lee ZH, Park J-C, Lee K-E, Lee M-H, Park J-T, Seo B-M, Hu JCC, Simmer JP (2008). FAM83H Mutations in Families with Autosomal-Dominant Hypocalcified Amelogenesis Imperfecta. American Journal of Human Genetics.

[86] Lee S-K, Hu JCC, Bartlett JD, Lee K-E, Lin BPJ, Simmer JP, Kim J-W (2008). Mutational Spectrum of FAM83H: The C-Terminal Portion is Required for Tooth Enamel Calcification. Human mutation.

[87] Lee S-K, Lee K-E, Jeong T-S, Hwang Y-H, Kim S, Hu JC-C, Simmer JP, Kim J-W (2011). FAM83H Mutations Cause ADHCAI and Alter Intracellular Protein Localization. Journal of Dental Research.

[88] Wang S-K, Hu Y, Yang J, Smith CE, Richardson AS, Yamakoshi Y, Lee Y-L, Seymen F, Koruyucu M, Gencay K, Lee M, Choi M, Kim J-W, Hu JCC, Simmer JP (2016). Fam83h null mice support a neomorphic mechanism for human ADHCAI. Molecular Genetics & Genomic Medicine.

[89] Kuga T, Kume H, Kawasaki N, Sato M, Adachi J, Shiromizu T, Hoshino I, Nishimori T, Matsubara H, Tomonaga T (2013). A novel mechanism of keratin cytoskeleton organization through casein kinase I and FAM83H in colorectal cancer. Journal of Cell Science.

[90] Oughtred R, Chatr-aryamontri A, Breitkreutz B-J, Chang CS, Rust JM, Theesfeld CL, Heinicke S, Breitkreutz A, Chen D, Hirschman J, Kolas N, Livstone MS, Nixon J, O'Donnell L, Ramage L, Winter A (2016). Use of the BioGRID Database for Analysis of Yeast Protein and Genetic Interactions. Cold Spring Harbor Protocols.

[91] Hornbeck PV, Zhang B, Murray B, Kornhauser JM, Latham V, Skrzypek E (2015). PhosphoSitePlus, 2014: mutations, PTMs and recalibrations. Nucleic Acids Research.

[92] Welcker M, Clurman BE (2008). FBW7 ubiquitin ligase: a tumour suppressor at the crossroads of cell division, growth and differentiation. Nat Rev Cancer.

[93] Dinkel H, Van Roey K, Michael S, Davey NE, Weatheritt RJ, Born D, Speck T, Krüger D, Grebnev G, Kuba M, Strumillo M, Uyar B, Budd A, Altenberg B, Seiler M, Chemes LB (2014). The eukaryotic linear motif resource ELM: 10 years and counting. Nucleic Acids Research.

[94] Nakayama KI, Nakayama K (2006). Ubiquitin ligases: cell-cycle control and cancer. Nat Rev Cancer.

[95] Obenauer JC, Cantley LC, Yaffe MB (2003). Scansite 2. 0: proteome-wide prediction of cell signaling interactions using short sequence motifs. Nucleic Acids Research.

[96] Nero TL, Morton CJ, Holien JK, Wielens J, Parker MW (2014). Oncogenic protein interfaces: small molecules, big challenges. Nat Rev Cancer.

[97] White CD, Brown MD, Sacks DB (2009). IQGAPs in Cancer: A Family of Scaffold Proteins Underlying Tumorigenesis. FEBS letters.

[98] Johnson M, Sharma M, Henderson BR (2009). IQGAP1 regulation and roles in cancer. Cellular Signalling.

[99] Jameson KL, Mazur PK, Zehnder AM, Zhang J, Zarnegar B, Sage J, Khavari PA (2013). IQGAP1 scaffold-kinase interaction blockade selectively targets RAS-MAP kinase–driven tumors. Nature medicine.

[100] Hao Y, Wang C, Cao B, Hirsch BM, Song J, Markowitz SD, Ewing RM, Sedwick D, Liu L, Zheng W, Wang Z (2013). Gain of interaction with IRS1 by p110 helical domain mutants is crucial for their oncogenic functions. Cancer cell.

[101] Rajalingam K, Rudel T (2005). Ras-Raf signaling needs prohibitin. Cell Cycle.

[102] Luan Z, He Y, Alattar M, Chen Z, He F (2014). Targeting the prohibitin scaffold-CRAF kinase interaction in RAS-ERK-driven pancreatic ductal adenocarcinoma. Molecular Cancer.

[103] Athuluri-Divakar Sai K, Vasquez-Del Carpio R, Dutta K, Baker Stacey J, Cosenza Stephen C, Basu I, Gupta Yogesh K, Reddy MVR, Ueno L, Hart Jonathan R, Vogt Peter K, Mulholland D, Guha C, Aggarwal Aneel K, Reddy EP A Small Molecule RAS-Mimetic Disrupts RAS Association with Effector Proteins to Block Signaling. Cell.

[105] Pinkas DM, Sanvitale C, Wang D, Krojer T, Kopec J, Chaikuad A, Dixon Clarke S, Berridge G, Burgess-Brown N, von Delft F, Arrowsmith C, Edwards A, Bountra C, Bullock A, PDB ID: 4URJ Crystal structure of human BJ-TSA-9.

[105] Zhou J, Shum K-T, Burnett JC, Rossi JJ (2013). Nanoparticle-Based Delivery of RNAi Therapeutics: Progress and Challenges. Pharmaceuticals.

[106] Wang X-L, Xu R, Wu X, Gillespie D, Jensen R, Lu Z-R (2009). Targeted Systemic Delivery of a Therapeutic siRNA with a Multifunctional Carrier Controls Tumor Proliferation in Mice. Molecular Pharmaceutics.

[107] Wang X-L, Xu R, Lu Z-R (2009). A peptide-targeted delivery system with pH-sensitive amphiphilic cell membrane disruption for efficient receptor-mediated siRNA delivery. Journal of Controlled Release.

[108] Malamas AS, Gujrati M, Kummitha CM, Xu R, Lu Z-R (2013). Design and Evaluation of New pH-Sensitive Amphiphilic Cationic Lipids for siRNA Delivery. Journal of controlled release.

[109] Young SWS, Stenzel M, Jia-Lin Y (2016). Nanoparticle-siRNA: A potential cancer therapy?. Critical Reviews in Oncology / Hematology.

[110] Parvani JG, Gujrati MD, Mack MA, Schiemann WP, Lu Z-R (2015). Silencing 3 Integrin by Targeted ECO/siRNA Nanoparticles Inhibits EMT and Metastasis of Triple-Negative Breast Cancer. Cancer Research.

